# Association between greenspace morphology and dengue fever in China

**DOI:** 10.1186/s13071-025-06727-w

**Published:** 2025-03-22

**Authors:** Yingying Cao, Wenhao Yu, Chuanxi Li, Zunyan Chu, Bangjie Guo, Haitao Wang, Wei Ma, Xueshui Xu, Qiyong Liu, Qi Zhao

**Affiliations:** 1 Department of Vector Control, School of Public Health, Cheeloo College of Medicine, Shandong University, Jinan, China; 2 Department of Epidemiology, School of Public Health, Cheeloo College of Medicine, Shandong University, Jinan, China; 3 Shandong University Climate Change and Health Centre, Shandong University, Jinan, China; 4 Qilu Hospital of Shandong University, Cheeloo College of Medicine, Shandong University, Jinan, China; 5 Dezhou Center for Disease Control and Prevention, Dezhou, China; 6 State Key Laboratory of Infectious Disease Prevention and Control, National Institute for Communicable Disease Control and Prevention, Chinese Centre for Disease Control and Prevention, Beijing, China; 7 Faculty of Health, Deakin University, Melbourne, Victoria 3000 Australia

**Keywords:** Greenspace morphology, Dengue, Urbanization, Built environments

## Abstract

**Background:**

Although the contribution of greenspace to dengue transmission has been reported, the complex role of greenspace morphology remains unclear. We aimed to investigate the relationship between greenspace morphology and dengue in China and to explore the interaction with urbanization and built environment characteristics.

**Methods:**

Dengue data at the township level were collected from five provinces in southern China during 2017–2020. Metrics of greenspace morphology, including percentage, mean area, fragmentation, shape, aggregation, and connectedness, were calculated to quantify its structural characteristics. A negative binomial regression model combined with principal component analysis was conducted to assess the relationship between greenspace morphology and dengue. The modification effects of urbanization and built environment characteristics were evaluated using an interaction term in the model.

**Results:**

Per-interquartile range increase in total percentage, mean area, area-weighted mean shape index, and aggregation index of greenspace were associated with 1.78 (95% confidence interval [CI] 1.57–2.01), 1.14 (1.10–1.20), 1.17 (1.06–1.29), and 1.18 (1.11–1.26) incidence rate ratios of dengue, respectively, while edge density was negatively related to the risk of dengue. In areas with high gross domestic product per capita and population size, the impact of greenspace morphology on the incidence of dengue was more pronounced. By contrast, the influence of greenspace morphology on dengue risk was diminished in regions characterized by higher urban isolation and fragmentation.

**Conclusions:**

Greenspace morphology had a bidirectional impact on the risk of dengue, with urbanization and built environment characteristics exerting diverse modification effects. Our study highlights the importance of a rational greenspace layout to prevent the spread of dengue.

**Graphical Abstract:**

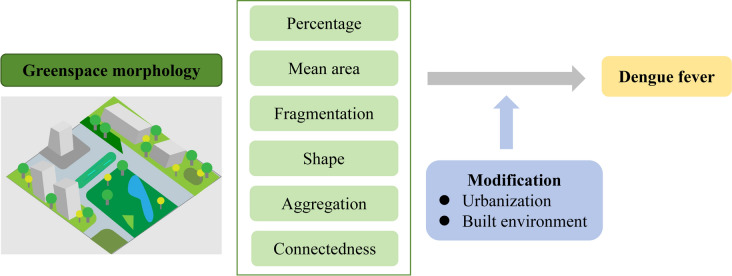

**Supplementary Information:**

The online version contains supplementary material available at 10.1186/s13071-025-06727-w.

## Background

Dengue is a mosquito-borne disease caused by the dengue virus and is transmitted to humans through the bite of the *Aedes* mosquito [1, 2]. According to the estimation of the World Health Organization, about half of the world’s population is at risk of dengue infection [3]. Over the past three decades, dengue has been spread extensively, affecting approximately 129 countries and territories [4]. In China, dengue fever has been rising as an important challenge for public health [5]: during 2005–2020, a total of 81,653 indigenous dengue cases were reported, with the distribution spreading from the southeastern coast at low latitudes to the central and northern regions at high latitudes [6].

In recent years, increasing attention has been directed towards the potential influence of urban greenspace, vegetation, and land cover on the risk of dengue transmission, as these factors may alter habitats and populations of mosquitoes [7, 8]. For example, abundant vegetation and water resources provided by greenspace create an ideal environment for mosquitoes [9]. Previous studies have explored the relationship between greenspace and the incidence of dengue fever, but the conclusions were inconsistent [7, 10–13]. One explanation may be that these studies focused mainly on the effect of the normalized difference vegetation index, with the complex role of specific spatial layout of greenspace neglected. The morphology of greenspace characterizes the greenspace area and reflects the spatial distribution and configuration of green patches in the area (e.g., the fragments, size distribution, shape, and spatial arrangement) [14]. Relevant studies have confirmed that the composition and morphology of greenspace landscapes is closely related to the risk of infectious diseases [15, 16]. Therefore, it is necessary to clarify the association between the spatial arrangement of greenspace and dengue fever to improve the prevention and control of dengue.

Additionally, the relationship between greenspace and dengue can be modified by specific urbanization and built environment characteristics [17]. Different accessibility of greenspace and environmental sanitation related to the local socioeconomic and urbanization levels may influence the spread of dengue indirectly [18, 19]. A Brazilian study discovered that the correlation between dengue and greenness was contingent on socioeconomic vulnerability. The risk of dengue was higher in areas with higher vulnerability and lower-quality greenspace [17]. Meanwhile, the spread of dengue fever may be influenced by the urban built environment, which is designed as a diverse, heterogeneous, fragmented space with extensive and rich social content [20]. However, evidence for the interaction between greenspace morphology and city development on the risk of dengue remains limited.

In this study, we aimed to explore the association between greenspace morphology and the incidence of dengue in China, and to investigate the modification effect of urbanization and built environment characteristics.

## Methods

### Study area

Five provinces in southern China (Zhejiang, Fujian, Guangdong, Guangxi, and Yunnan) were selected as the study area, where local dengue cases accounted for 85.5% of cases nationwide from January 1, 2017, to December 31, 2020. The study area is located at 97°31E to 123°10E and 20°09N to 31°11N, with an area of 1.0441 million km^2^ and resident population of 309 million. There are 6733 streets (1361 with dengue cases reported) in the five provinces; streets without detailed street information and identified greenspaces were excluded in the current study.

### Dengue fever cases

Dengue fever has been classified as a notifiable infectious disease (category B) in China since 1989 and is subject to statutory reporting [21]. Data on dengue fever cases at the township level in the five provinces between January 1, 2017, and December 31, 2020, were collected from the national notifiable infectious disease surveillance system of the Chinese Center for Disease Control and Prevention. Imported and suspected or unconfirmed cases were not included in this study. All cases were diagnosed clinically and confirmed in the laboratory by professional medical institutions, and the specific diagnostic criteria are shown in the Supporting Text of the appendix.

### Exposure assessment

The morphology of greenspace at the township level was quantified based on the annual land use/land cover dataset (with a resolution of 10 m × 10 m) from the Environmental Systems Research Institute (Esri) from 2017 to 2020 [22]. This dataset contains nine categories, with trees and rangeland defined as greenspace. Consistent with previous studies, the area and edge, shape, and aggregation were considered as exposure measurements in this study [23, 24]. Six landscape metrics (Additional file: Table S1) representing greenspace morphology, including percentage of landscape, mean patch area, edge density, area-weighted mean shape index, aggregation index, and patch cohesion index, were calculated [25].

We selected potential confounders through an extensive search of previous literature. Climate data at the township level were extracted from the fifth-generation European Centre for Medium-Range Weather Forecasts reanalysis of the global climate (ERA5) monthly averaged data, a global gridded satellite dataset with a resolution of 0.25° × 0.25° [26]. Information about demographic (population size) and socioeconomic factors [gross domestic product (GDP) per capita] and built environment characteristics (urban isolation and urban fragmentation) were also included. Population data for each street between 2017 and 2020 were extracted from the WorldPop database [27]. Raster data for GDP were obtained from a modeling study with a spatial resolution of 1 km × 1 km [28]. The built environment characteristics came from the annual land use/land cover dataset of Esri and were calculated based on the area of the built area [22]. Urban isolation was calculated by dividing the sum of the nearest-neighbor distances of patches in the area by the number of patches. Urban fragmentation was derived by dividing the total number of patches by the area of the 10 m × 10 m grid cell.

### Statistical analysis

Negative binomial regression models were used to examine the relationship between each landscape metric and dengue cases. A univariate linear regression model was used to determine variables that were significantly associated with dengue fever. At the same time, variables with high correlation were excluded according to the correlation coefficients (|*r*_s_|> 0.7) and variance inflation factor (VIF ≥ 3). In the main model, we controlled for average annual temperature, cumulative annual rainfall, relative humidity, total population, GDP per capita, and urban isolation. The results of the models are reported as incidence rate ratios (IRRs) and 95% confidence intervals (CIs). Principal component analysis was implemented to consider the distribution across the greenspace landscape as a whole. The effects of principal components with eigenvalues greater than 1 on the risk of dengue were also examined. To assess the modification effect of urbanization and built environment characteristics on the association between greenness structure indices and the incidence of dengue fever, a linear interaction term between greenness structure indices and urban development indicators was included in the main model. Each urban development indicator (GDP per capita, population, urban isolation, and urban fragmentation) was stratified into high (> 75th percentile of overall distribution) and low (≤ 25th percentile of overall distribution) levels.

Sensitivity analyses were performed by changing the covariates of the main model to test the robustness of the results. The R package “MASS” was used to build the models [29]. A *P*-value < 0.05 was considered statistically significant.

## Results

### Characteristics of dengue cases and greenspace morphology

**Fig. 1:**
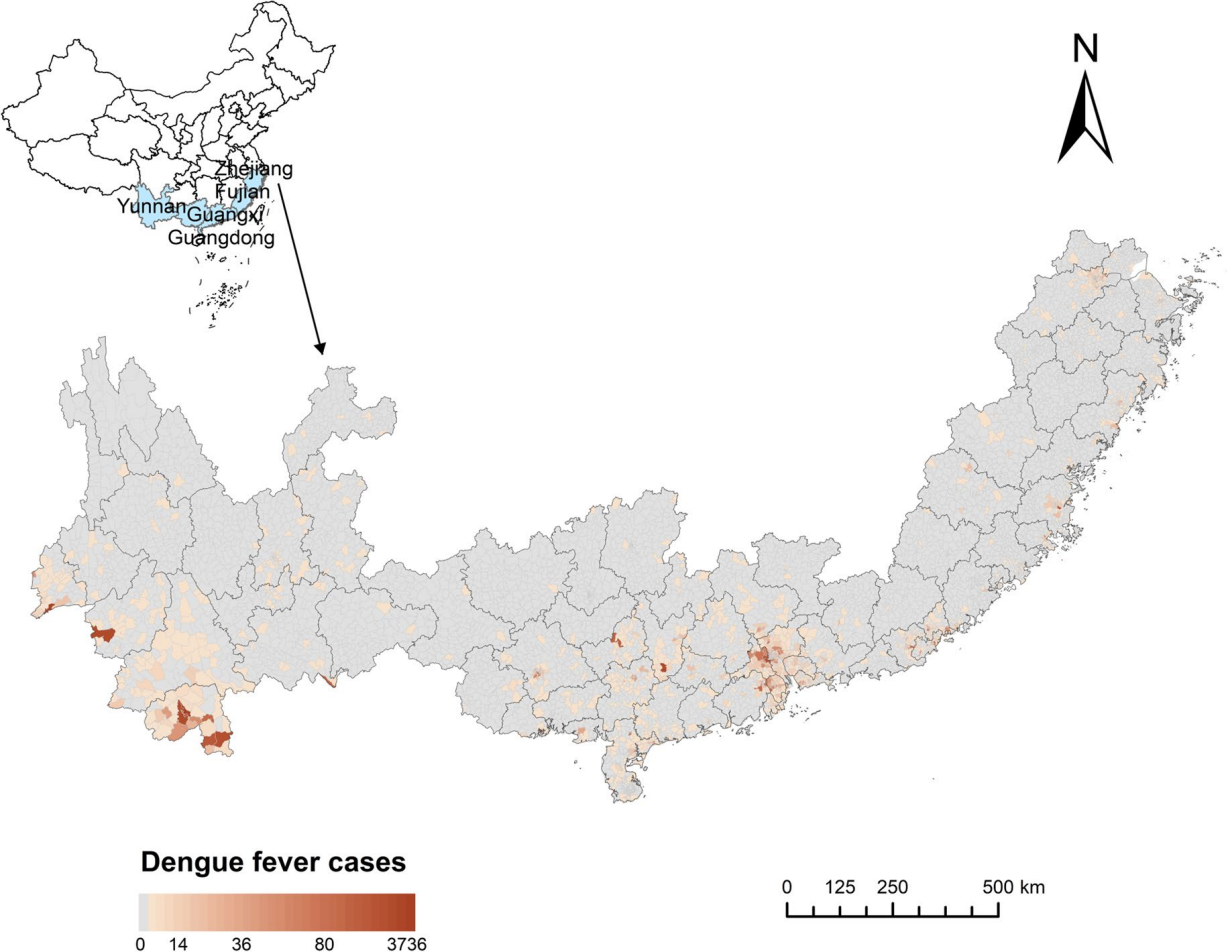
Geographical locations and spatial distribution of dengue fever cases for the five provinces in China from 2017 to 2020

A total of 19,252 local dengue cases were reported from 1198 streets in five provinces during the study period. The geographical distribution of dengue cases is shown in Fig. 1. Yunnan Province has the highest cumulative number of cases (7898 cases) and cumulative incidence rate of dengue (16.8 cases per 100,000 people). The annual average temperature, cumulative rainfall, and relative humidity in the study area during 2017–2020 were 19.8 °C, 1633.2 mm, and 76.4%, respectively (Additional file 1: Figure S1 and Table S2). The characteristics of the greenspace morphology were determined for each township (Table 1).

**Table 1:** Characteristics of landscape distribution in the study area

Variables	Mean (SD)	First Quartile	Median	Third Quartile
Percentage of landscape	35.5 (32.3)	4.97	24.6	64.4
Mean patch area	67.3 (166)	2.72	14.2	61.1
Edge density	18.2 (11.5)	9.38	16.6	25.1
Area-weighted mean shape index	4.14 (2.83)	1.97	3.33	5.37
Aggregation index	96.2 (4.22)	94.5	97.7	99.0
Patch cohesion index	97.6 (4.31)	96.9	99.4	99.9

### Impact of greenspace morphology on dengue fever

**Fig. 2:**
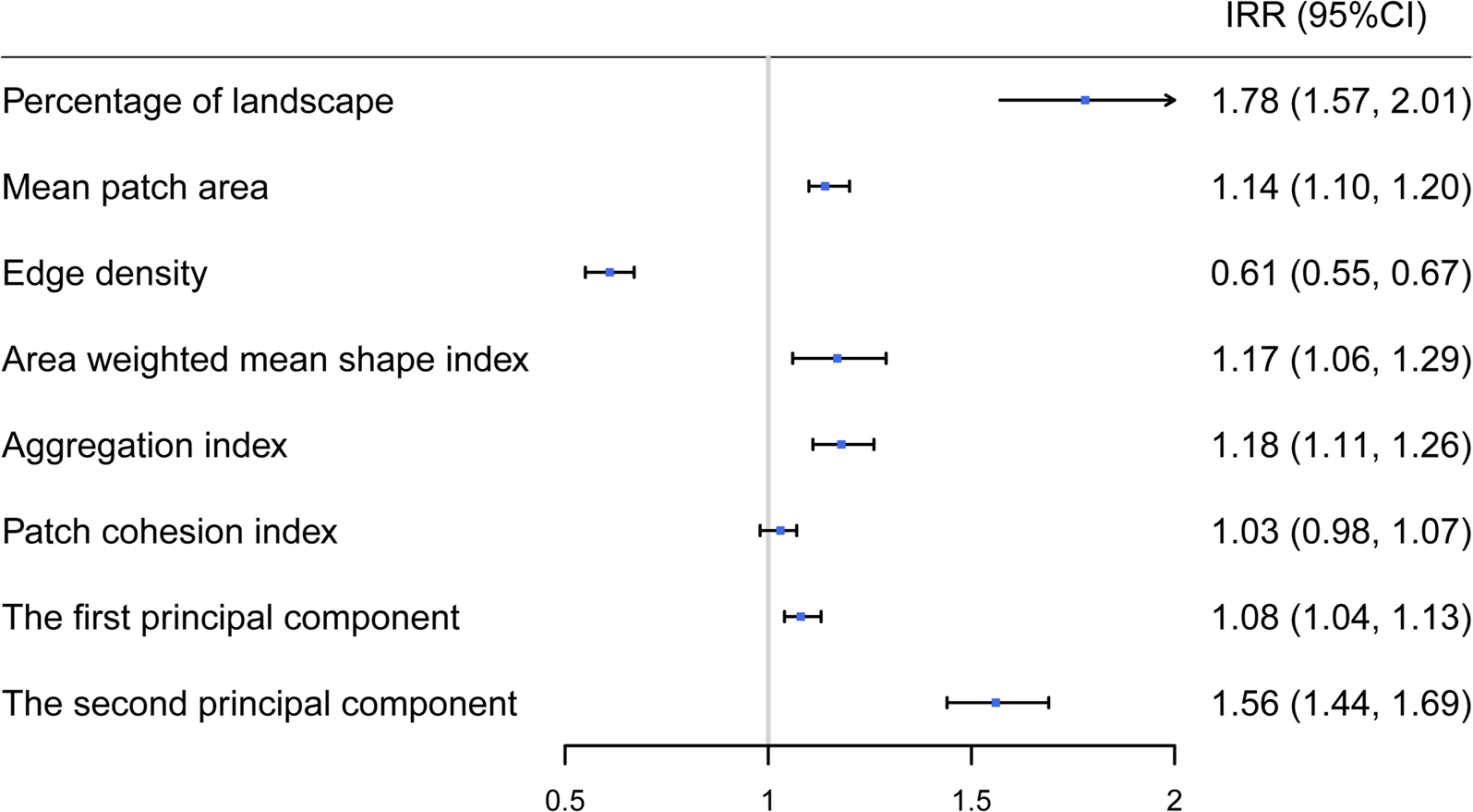
Association between greenness structure indices (per interquartile range increase) and dengue fever cases. IRR, incidence rate ratio; CI, confidence interval

As shown in Fig. 2 and Additional file: Table S3, areas with a higher percentage of greenspace were associated with a higher risk of dengue, such that for each interquartile range (IQR) increase in percentage of greenspace, the number of dengue cases was expected to increase 77.5% (IRR, 1.78; 95% CI 1.57–2.01). In addition, every increase in mean area, area-weighted mean shape index, and aggregation index of greenspace was associated with increased risk of dengue fever, with IRRs of 1.14 (95% CI 1.10–1.20), 1.17 (1.06–1.29), and 1.18 (1.11–1.26), respectively. By contrast, edge density was negatively correlated with the number of dengue cases (IRR, 0.61; 95% CI 0.55–0.67). There was no significant relationship between greenfield connectivity and dengue. The first two principal components, with eigenvalues exceeding 1, cumulatively accounted for 75.2% of the total variance (Additional file: Figure S2 and Table S4). The contribution of each landscape metric on the first principal component was relatively homogeneous, with factor loadings ranging from 0.261 to 0.482. On the second principal component, there was a higher factor loading for the mean area and edge density of greenspace (Additional file: Table S5). The landscape distribution of greenspace was positively correlated with dengue cases as a whole (Fig. 2).

### Modification effects of urbanization and built environment characteristics

**Fig. 3:**
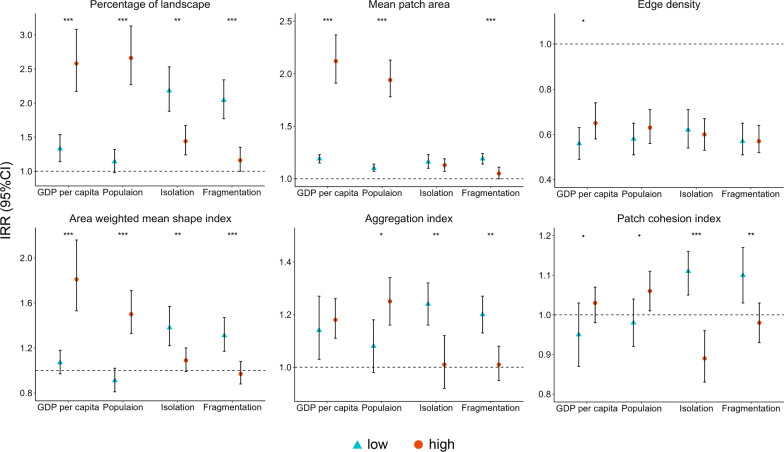
Incidence rate ratio of dengue for greenness structure indices per interquartile range increase at different levels of urbanization and built environment characteristics, *P* < 0.1 for interaction. *, *P* < 0.05 for interaction. **, *P* < 0.01 for interaction. ***, *P* < 0.001 for interaction. Low, low level of urbanization and built environment characteristics (concentration for 25th value); high, high level of urbanization and built environment characteristics (concentration for 75th value). Isolation, mean distance to the nearest urban patch within the geographical boundary; fragmentation, number of urban patches divided by the total area of the geographical unit; IRR, incidence rate ratio; CI, confidence interval

Figure 3 shows the relationship between the greenness structure indices and the IRR of dengue by different urbanization and built environment characteristics. In areas with high GDP per capita and population size, the impact of greenspace morphology on the occurrence of dengue fever was greater. For example, the IRR of dengue was 2.58 (95% CI 2.17–3.08) for each IQR increase in the percentage of greenspace in areas with high GDP per capita, and 1.33 (95% CI 1.14–1.54) in those with low levels (Fig. 3 and Additional file: Table S6). Similar patterns were found for other greenness structure indices, though the differences in the effect of edge density and patch cohesion index on the risk of dengue between high and low levels of urbanization were not statistically significant. Conversely, the influence of the greenness structure indices on the occurrence of dengue fever was diminished in areas with high levels of urban isolation and fragmentation. For example, the IRR of dengue under high and low levels of urban isolation was projected to be 1.44 versus 2.18 for percentage of greenspace, 1.09 versus 1.38 for area-weighted mean shape index, and 1.01 versus 1.24 for aggregation index, respectively (Fig. 3 and Additional file: Table S6). Differences in the effects of edge density and mean area of greenspace on dengue risk were not statistically significant between different levels of urban isolation.

Sensitivity analyses indicated that our results were robust by changing the covariates of the main model (Additional file: Table S7).

## Discussion

In this study, we explored the relationship between greenspace landscape morphology and the risk of dengue, and further investigated the modification effect of urbanization and built environment characteristics on this association. Our findings indicated that areas with a larger total percentage of greenspace, mean area, area-weighted mean shape index, and aggregation index, and smaller edge density were associated with a higher risk of dengue. Urbanization and built environment characteristics modified the effect size of greenspace morphology in different patterns.

Previous studies suggest that there are several potential explanations for our finding of a positive association between the total percentage of greenspace, mean area of greenspace, and the risk of dengue. Greenspace might contribute to an increase in dengue by influencing both mosquito and human behavior [23, 30, 31]. For example, larger greenspace implies more mosquito populations and the presence of more vegetation and water, increasing the possibility of dengue transmission [31]. Poorly managed environments in large greenspaces, characterized by water accumulation and garbage retention, could add opportunities for mosquito breeding. A Chinese study found that people preferred to spend time in greenspace with larger size, which was designed to attract people for health-related activities [24].

In addition, we found that complex shapes of greenspace were also linked to a higher risk of dengue. This can be attributed to the impact of greenspace shape on the survival of vectors. Irregular forest remnants have been linked to a greater abundance of mosquitoes [32]. In addition, greenspace with complex shapes provides more access points, increasing the willingness of people to engage in greenspace [23]. A study conducted in Africa found that optimal geometry of habitats played a crucial role in minimizing the risk of infectious diseases, as it determines the contact areas where disease transmission may occur [33]. Emphasizing simplicity rather than complex or irregular patch geometries will reduce the range of interaction between humans and vectors.

Our findings indicated a positive correlation between the greenspace aggregation index and the risk of dengue. A previous study similarly highlighted the need to control aggregation levels in grasslands and water bodies in order to reduce the potential transmission of West Nile virus [34]. This may be due to the fact that residents are prompted to interact with natural elements more frequently in aggregated greenspaces [23]. Certain vectors, such as *Aedes aegypti* and *Aedes albopictus* with limited capacity for dispersal and migration, can transmit pathogens over short distances only [35, 36]. As a result, habitats characterized by congregation may be more conducive to the transmission of dengue fever.

Moreover, we observed a positive trend, although not statistically significant, in the relationship between greenspace connectivity and dengue. More human activity in highly connected greenspaces increases the contact between people and mosquitoes and thereby elevates the risk of dengue infection. Interconnected greenspaces not only provide more space for leisure and outdoor activities, but also make it easier for people to move between different greenspaces [23]. In addition, the risk of transmission of mosquito-borne infectious diseases such as dengue fever also depends on the connectivity of habitats and the spatial dispersal of vectors. The importance of connectivity between habitats for the spread of mosquitoes was also demonstrated in a study focused on *Culex* species in southern France [37]. Previous research has suggested that the risk of vector-borne diseases would increase if critical habitats for mosquito vectors were connected through landscape features [38].

By contrast, we found that edge density was inversely correlated with dengue risk. Edge density is used to reveal the degree to which the landscape is divided by boundaries, as well as the degree of greenspace boundary fragmentation [39]. A large number of studies have identified habitat fragmentation as a significant threat to biodiversity [40, 41]. Habitat fragmentation was found to decrease both the size and persistence of mosquito populations [42]. As a kind of obligate semiaquatic insect, the presence and reproduction of mosquitoes in specific areas is regulated by the availability of suitable larval habitat. In addition, fragmented habitat edges are more susceptible to environmental disturbances such as human activities and climatic factors [43]. These disturbances may alter the habitat of mosquitoes and worsen their breeding and survival conditions. However, some studies have indicated that fragmentation of land use was associated with increased interactions between humans and mosquitoes, leading to the spread of pathogens [44, 45]. For example, a study in Colombia reported a positive correlation between the linear density of cropland and grassland and Zika virus risk [46]. Further research is needed to clarify the exact mechanisms underpinning the relationship between greenspace fragmentation and the risk of dengue transmission.

The results of this study suggest that urbanization and built environment characteristics have important modification effects on the association between greenspace and dengue risk. The impact of greenspace on the incidence of dengue fever was stronger in areas with high GDP per capita and population levels. Williams et al. found that in economically developed regions, individuals possessed more leisure time and disposable income, making them more inclined to partake in outdoor activities and utilize greenspaces [47]. Meanwhile, these regions tended to prioritize environmental conservation and urban greening [48], further increasing people’s exposure to mosquitoes. In densely populated areas, the heightened probability of mosquito bites increases the risk of dengue virus transmission. Additionally, our findings indicated that in regions characterized by higher levels of urban isolation and fragmentation, the impact of greenspace on dengue risk was diminished. Individuals exhibited lower mobility and tended to stay within specific areas in highly segregated and fragmented urban environments [49]. This limited mobility reduces the possibility of dengue transmission.

This study has several strengths. First, this is the first study to examine the relationship between greenspace morphology and the risk of dengue. The high coverage of areas with high risk of dengue fever in mainland China ensures the representativeness of the findings. Second, we explored the modification effects of urbanization and built environment characteristics on the greenspace–dengue association. These findings suggest that urban greenspace planning plays a crucial role in controlling infectious diseases such as dengue fever, providing a foundation for the formulation of relevant prevention and control measurements. Third, high-resolution (10 m) data used in this study ensured the reliability of our results.

Some limitations should be acknowledged. This study period lasted only 4 years due to time constraints on dengue data and land use data, which might limit our ability to capture long-term trends in dengue epidemics and greenspace morphological characteristics. This limitation may be solved in the future when more data are available. Additionally, there is a lack of methods to measure individual exposure, which may bias the association between greenspace morphology and dengue fever. To address the potential exposure measurement bias, detailed data on individual greenspace exposure should be collected through the combined use of surveys, wearable sensors, and mobile applications in the future. In addition, information on mosquito abundance was not included in the current analysis because of the limited data availability. Finally, we are not authorized to access information on the serotypes and clinical forms of dengue infections, which may preclude a more comprehensive analysis and understanding of the disease. Efforts should be made to collect relevant data by collaborating with local entomological and public health authorities, to investigate how greenspace directly affects mosquito ecology and to better understand the dynamics of dengue fever.

Future research perspectives may be necessary. Although this study explored the impact of greenspace morphology on dengue transmission, in-depth research is still needed to understand how the morphological characteristics of different types of greenspace (parks, woodlands, grasslands, etc.) affect dengue transmission. The findings could provide more specific guidance for the management and planning of urban greenspaces. In addition, current research focused mainly on the short-term effects of greenspace morphology. The long-term variation in the association between greenspace morphology and dengue incidence remains unclear. Clarification of this research question may enable a better understanding of the dynamics of dengue ecology. Finally, integration of mosquito abundance data is recommended in order to understand the role of greenspace morphology on mosquito populations.

## Conclusions

Our findings showed that the bidirectional impacts of greenspace on dengue or other vector-borne diseases should be considered when formulating urban greenspace planning policies to achieve the maximum ecological and social benefit. It is particularly important to strengthen the regular management of large greenspaces. In addition, small-area greenspaces could be developed to reduce the size and aggregation levels of greenspaces. The morphological complexity of greenspaces should also be controlled for to optimize their spatial patterns.

## Supplementary Information

Additional file 1. Diagnosis for dengue in mainland China. Table S1. Description of landscape metrics used in this study. Table S2. Descriptive statistics of climatic, urbanization, and built environment characteristics in the study area, 2017–2020. Table S3. Percentage change in dengue cases for greenness structure indices per interquartile range increase. Table S4. Descriptive statistics of original principal components. Table S5. Factor loadings of the principal component analysis. Table S6. Incidence rate ratio of dengue for greenness structure indices per interquartile range increase at different levels of urbanization and built environment characteristics. Table S7. Sensitivity analyses by changing covariates in the main model. Figure S1. Spatial distribution of annual mean value of climatic, urbanization, and built environment characteristics of the five provinces in China, 2017–2020. Figure S2. Gravel diagram of principal components of greenspace component.

## Data Availability

No datasets were generated or analyzed during the current study.
