# Supplementary material for: Association between greenspace morphology and dengue fever in China
Source: Parasit Vectors. 2025 Mar 22;18:115. doi: 10.1186/s13071-025-06727-w (PMC11929997; doi:10.1186/s13071-025-06727-w)
Supplement: Supplementary file 1 — Additional file 1. Diagnosis for dengue in mainland China. Table S1. Description of landscape metrics used in this study. Table S2. Descriptive statistics of climatic, urbanization, and built environment characteristics in the study area, 2017–2020. Table S3. Percentage change in dengue cases for greenness structure indices per interquartile range increase. Table S4. Descriptive statistics of original principal components. Table S5. Factor loadings of the principal component analysis. Table S6. Incidence rate ratio of dengue for greenness structure indices per interquartile range increase at different levels of urbanization and built environment characteristics. Table S7. Sensitivity analyses by changing covariates in the main model. Figure S1. Spatial distribution of annual mean value of climatic, urbanization, and built environment characteristics of the five provinces in China, 2017–2020. Figure S2. Gravel diagram of principal components of greenspace component. [file 13071_2025_6727_MOESM1_ESM.docx]

**Supporting information**

**Association between greenspace morphology and dengue fever in China.**

**Yingying Cao^1,2,†^,** **Wenhao Yu^2,3,†^, Chuanxi Li^4^,** **Zunyan Chu^2,3^, Bangjie Guo^2,3^, Haitao Wang^2,3^, Wei Ma^2,3^, Xueshui Xu^5*^, Qiyong Liu^1,2,3,6*^, Qi Zhao^2,3,7*^**

**Affiliations:**

^1^Department of Vector Control, School of Public Health, Cheeloo College of Medicine, Shandong University, Jinan, China;

^2^Department of Epidemiology, School of Public Health, Cheeloo College of Medicine, Shandong University, Jinan, China;

^3^Shandong University Climate Change and Health Centre, Shandong University, Jinan, China;

^4^Qilu Hospital of Shandong University, Cheeloo College of Medicine, Shandong University, Jinan, China;

^5^Dezhou Center for Disease Control and Prevention, Dezhou, China;

^6^State Key Laboratory of Infectious Disease Prevention and Control, National Institute for Communicable Disease Control and Prevention, Chinese Centre for Disease Control and Prevention, Beijing, China;

^7^Faculty of Health, Deakin University, Melbourne, Victoria, 3000, Australia.

^†^ Yingying Cao and Wenhao Yu contributed equally to this work.

**Correspondence author:**

Qi Zhao, PhD

Department of Epidemiology,

School of Public Health,

Shandong University Climate Change and Health Centre,

Cheeloo College of Medicine,

Shandong University,

Jinan, Shandong, 250012, China

E-mail: [qi.zhao@sdu.edu.cn](mailto:qi.zhao@sdu.edu.cn)

Qiyong Liu, PhD

State Key Laboratory of Infectious Disease Prevention and Control,

National Institute for Communicable Disease Control and Prevention,

Chinese Centre for Disease Control and Prevention,

Beijing 102206, China

E-mail: [liuqiyong@icdc.cn](mailto:liuqiyong@icdc.cn)

Xueshui Xu, BS

Dezhou Center for Disease Control and Prevention,

Dezhou, Shandong, 253012, China

E-mail: [xuxuesh@163.com](mailto:xuxuesh@163.com)

**Contents**

**1. Supporting Text**

[Diagnosis for dengue in mainland China 4](#_Toc188818698)

**2. Supporting Tables**

[Table S1. Description of landscape metrics used in this study. 7](#_Toc188818699)

[Table S2. Descriptive statistics of climatic, urbanization, and built environment characteristics in the study area, 2017-2020. 8](#_Toc188818700)

[Table S3. Percentage change in dengue cases for greenness structure indices per interquartile range increase. 9](#_Toc188818701)

[Table S4. Descriptive statistics of original principal components. 10](#_Toc188818702)

[Table S5. Factor loadings of the principal component analysis. 11](#_Toc188818703)

[Table S6. Incidence rate ratio of dengue for greenness structure indices per interquartile range increase at different levels of urbanization and built environment characteristics. 12](#_Toc188818704)

[Table S7. Sensitivity analyses by changing covariates in the main model. 14](#_Toc188818705)

**3. Supporting Figures**

[Figure S1. Spatial distribution of annual mean value of climatic, urbanization, and built environment characteristics of the five provinces in China, 2017-2020. 15](#_Toc188818706)

[Figure S2. Gravel diagram of principal components of greenspace component. 16](#_Toc188818707)

[References 17](#_Toc188818708)

**1. Supporting Text**

# Diagnosis for dengue in mainland China

During the study period (2017-2020), dengue fever was diagnosed based on the “Diagnostic criteria for dengue fever (WS 216-2008)” and “Diagnostic criteria for dengue fever (WS 216-2018)” issued by the National Health Commission. Diagnostic criteria for dengue fever (WS 216-2008) are as follows[1].

**1 Diagnostic basis**

1.1 Epidemiological history

1.1.1 Individuals have been to the dengue epidemic area within 14 days before the onset of the disease.

1.1.2 There was (were) dengue case(s) within 1 month around the living place or workplace (within a circle with radius of 100m).

1.2 Clinical manifestations

1.2.1 Acute onset with symptoms such as fever, headache, orbital pain, muscle pain, bone and joint pain, and obvious fatigue, or accompanied by facial, neck, chest flushing, conjunctival congestion, etc.

1.2.2 Rash: It appears as multiple rash (measles like, scarlet fever like, needle tip like, etc.) or “skin

island” like appearance on the 5^th^ to 7^th^ day of the course of disease. The rash is distributed on the trunk of the limbs or head and face, with itching and no desquamation, lasting for 3-5 days.

1.2.3 Bleeding tendency (tourniquet test positive): There is ecchymosis, ecchymosis, purpura, bleeding at the injection site, gingival bleeding, nasal bleeding and other mucosal bleeding, gastrointestinal bleeding, haemoptysis, haematuria, and vaginal bleeding on the 5^th^ to 8^th^ day.

1.2.4 Massive gastrointestinal bleeding, thoracic and abdominal bleeding, or intracranial haemorrhage.

1.2.5 Liver enlargement, hydrothorax, and ascites.

1.2.6 Shock symptoms such as wet and cold skin, irritability, hypotension, pulse pressure<20mmHg

(2.7kPa), blood pressure undetectable, or decreased urine volume.

1.3 Laboratory tests

1.3.1 Low white blood cell count.

1.3.2 Thrombocytopenia (<100×10^9^/L).

1.3.3 Blood concentration, such as increase of haematocrit by over 20% or hypoalbuminemia.

1.3.4 Specific IgG or IgM antibody is positive in any serum sample.

1.3.5 Dengue virus is isolated from serum, cerebrospinal fluid, blood cells, or tissues of patients in acute phase.

1.3.6 The titre of serum specific IgG antibody in the recovery phase is over 4 times higher than that in the acute phase.

1.3.7 Gene sequence of dengue virus is detected by RT-PCR or real-time fluorescent quantitative PCR.

**2 Diagnostic principle**

Dengue should be diagnosed comprehensively based on the epidemiological information, clinical

manifestations, and laboratory examination results.

**3 Diagnostic criteria**

3.1 Suspected cases

Meet one of the following conditions:

3.1.1 Meet 1.1.1 and 1.2.1.

3.1.2 Meet 1.2.1, 1.3.1, and 1.3.2.

3.2 Clinical diagnosis cases

3.2.1 Dengue fever

Meet one of the following conditions:

3.2.1.1 Meet 1.1.2, 1.3.1, and 1.3.2 in suspected cases.

3.2.2.2 Meet 3.1.2, and 1.3.4.

3.2.2 Dengue haemorrhagic fever

Meet 3.2.1, 1.3.2, 1.3.3, and one of 1.2.3, 1.2.4, and 1.2.5.

3.2.3 Dengue shock syndrome

Meet 3.2.2 and 1.2.6.

3.3 Confirmed cases

Meet one of 3.2 and one of 1.3.5, 1.3.6, and 1.3.7.

The updated criteria (criteria number: WS 216-2018) are as follows[2].

**1 Diagnostic basis**

1.1 Epidemiological history

The individuals have been to the dengue epidemic area within 14 days before the onset of the disease,

or there was (were) dengue case(s) occurred within 1 month around the living place or workplace.

1.2 Clinical manifestations

1.2.1 Acute onset, sudden high fever, obvious fatigue, anorexia, nausea, etc., often accompanied by

severe headache, orbital pain, muscle pain, bone and joint pain, or accompanied by facial, neck, chest flushing, conjunctival congestion, etc.

1.2.2 Rash: Hyperaemic rash or punctate haemorrhagic rash appears on the face and extremities on the 3^rd^ to 6^th^ day of the course of disease. Typical rashes are needle point-like bleeding spots in limbs and “skin island” like appearance. The rash is distributed on the trunk of the limbs or head and face, with itching and no desquamation, lasting for 3-5 days.

1.2.3 Bleeding tendency: Some patients may have bleeding manifestations of different degrees, such as subcutaneous bleeding, ecchymosis at the injection site, gingival bleeding, epistaxis, or tourniquet test positive.

1.2.4 Serious bleeding: Subcutaneous hematoma, gross haematuria, bleeding in digestive tract, chest and abdominal cavity, vagina, intracranial part, etc.

1.2.5 Serious organ injury: Acute myocarditis, acute respiratory distress syndrome, acute liver injury,

acute renal insufficiency, central nervous system injury, etc.

1.2.6 Shock: Tachycardia, limb dampness, the capillary filling time extended over 3 seconds, weak or undetectable pulse, decreased pulse pressure difference or undetectable blood pressure, etc.

1.3 Laboratory inspection

1.3.1 Low white blood cell and/or platelet count.

1.3.2 IgM antibody for dengue virus is positive.

1.3.3 Detection of NS1 antigen for dengue virus is positive within 5 days after the onset of disease.

1.3.4 The titre of serum specific IgG antibody in the recovery phase is over 4 times higher than that in the acute phase, or change from negative to positive.

1.3.5 Dengue virus is isolated from blood, cerebrospinal fluid, or tissue of patients in acute phase.

1.3.6 Nucleic acid of dengue virus is detected by RT-PCR or real-time fluorescent quantitative PCR.

**2 Diagnostic principle**

Dengue should be diagnosed comprehensively based on the epidemiological information, clinical

manifestations, and laboratory examination results.

**3 Diagnostic criteria**

3.1 Suspected cases

Meet one of the following conditions:

a) Meet 1.1 and 1.2.1.

b) Meet 1.2.1 and 1.3.1.

3.2 Clinical diagnosis cases

Meet one of the following conditions:

a) Meet 3.1a) and one of 1.2.2, 1.2.3, and 1.3.1.

b) Meet 3.1 and one of 1.3.2 and 1.3.3.

3.3 Confirmed cases

Meet 3.1 or 3.2, and one of 1.3.4, 1.3.5, and 1.3.6.

**2. Supporting Tables**

# Table S1. Description of landscape metrics used in this study.

| Landscape metrics | Abbreviation | Description | Range | Units |
| --- | --- | --- | --- | --- |
| Percentage of landscape | PLAND | PLAND equals the sum of the areas (m^2^) of all patches of the corresponding patch type, divided by total landscape area (m^2^), multiplied by 100 (to convert to a percentage). | 0 < PLAND ≤ 100  The higher the value, the higher the proportion of greenspace in the total area. | Percent |
| Mean patch area | AREA_MN | The metric summarizes the landscape as the mean of all patch in the landscape. | AREA_MN > 0  The higher the value, the larger the average area of greenspace. | Hectares |
| Edge density | ED | ED equals the sum of the lengths (m) of all edge segments involving the corresponding patch type, divided by the total landscape area (m^2^), multiplied by 10,000 (to convert to hectares). | ED ≥ 0, without limit.  ED = 0 when there is no class edge in the landscape.  The larger the value, the higher the boundary density of the greenspace. | Meters per hectare |
| Area weighted mean shape index | SHAPE_AM | The area-weighted mean shape index of patches at class and landscape levels was calculated by weighting patches according to their size. Specifically, larger patches are weighted more heavily than smaller patches in calculating the average patch shape for the class or landscape. | SHAPE ≤ 1, without limit. SHAPE = 1 when the patch is square and increases without limit as patch shape becomes more irregular.  The higher the value, the more irregular the shape of the greenspace. | Units |
| Aggregation Index | AI | AI equals the number of like adjacencies involving the corresponding class, divided by the maximum possible number of like adjacencies involving the corresponding class, which is achieved when the class is maximally clumped into a single, compact patch; multiplied by 100 (to convert to a percentage). | 0 ≤ AI ≤ 100  The higher the value, the higher the concentration of greenspace patches. | Percent |
| Patch Cohesion Index | COHESION | COHESION equals 1 minus the sum of patch perimeter (in terms of number of cells) divided by the sum of patch perimeter times the square root of patch area (in terms of number of cells) for all patches in the landscape, divided by 1 minus 1 over the square root of the total number of cells in the landscape, multiplied by 100 to convert to a percentage. | The behavior of this metric at the landscape level has not yet been evaluated.  The higher the value, the better the connectivity of greenspace patches. | None |

# Table S2. Descriptive statistics of climatic, urbanization, and built environment characteristics in the study area, 2017-2020.

| Variables | Mean (SD) | 1st Quartile | Median | 3rd Quartile |
| --- | --- | --- | --- | --- |
| **Meteorological conditions** |  |  |  |  |
| Temperature (℃) | 19.8 (2.87) | 18.1 | 19.8 | 22.2 |
| Precipitation (mm) | 1630 (323) | 1430 | 1590 | 179 |
| Relative humidity (%) | 76.4 (4.07) | 74.4 | 76.9 | 79.0 |
| **Urbanization indicators** |  |  |  |  |
| GDP per capita (10,000 yuan) | 5.88 (13.9) | 0.374 | 2.25 | 7.15 |
| Population (1000) | 46.3 (59.5) | 13.4 | 28.1 | 57.1 |
| **Built environment characteristics** |  |  |  |  |
| Urban isolation | 12.8 (7.88) | 6.73 | 12.2 | 17.8 |
| Urban fragmentation | 4.39 (4.99) | 1.44 | 2.76 | 5.37 |

# Table S3. Percentage change in dengue cases for greenness structure indices per interquartile range increase.

| Landscape Metrics | Percent Change | 95%CI |
| --- | --- | --- |
| Percentage of landscape | 77.5 | (56.9, 101) |
| Edge density | 14.4 | (9.69, 19.5) |
| Mean patch area | -39.4 | (-45.3, -32.9) |
| Area weighted mean shape index | 16.8 | (6.17, 28.6) |
| Patch cohesion index | 18.5 | (10.8, 26.3) |
| Aggregation index | 2.93 | (-1.69, 7.40) |

CI, confidence interval.

# Table S4. Descriptive statistics of original principal components.

|  | PC1 | PC2 | PC3 | PC4 | PC5 | PC6 |
| --- | --- | --- | --- | --- | --- | --- |
| Eigenvalue | 3.28 | 1.23 | 0.762 | 0.404 | 0.187 | 0.134 |
| Proportion | 0.547 | 0.205 | 0.127 | 0.067 | 0.031 | 0.022 |
| Cumulative | 0.547 | 0.752 | 0.879 | 0.947 | 0.978 | 1.00 |

PC, principal components; Proportion, proportions of variance; Cumulative, cumulative proportion.

# Table S5. Factor loadings of the principal component analysis.

| Variable | Comp 1 | Comp 2 | Comp 3 | Comp 4 | Comp 5 | Comp 6 |
| --- | --- | --- | --- | --- | --- | --- |
| Percentage of landscape | 0.482 | 0.263 | 0.239 | 0.175 | 0.541 | 0.565 |
| Edge density | 0.298 | -0.616 | 0.319 | -0.618 | 0.195 | -0.098 |
| Mean patch area | 0.261 | 0.712 | 0.051 | -0.584 | -0.191 | -0.212 |
| Area weighted mean shape index | 0.437 | 0.047 | 0.561 | 0.441 | -0.474 | -0.271 |
| Patch cohesion index | 0.449 | -0.203 | -0.519 | -0.086 | -0.521 | 0.457 |
| Aggregation index | 0.467 | 0.037 | -0.504 | 0.211 | 0.370 | -0.587 |

|  | Percentage of landscape | | Mean patch area | | Edge density | | Area weighted mean shape index | | Aggregation index | | Patch cohesion index | |
| --- | --- | --- | --- | --- | --- | --- | --- | --- | --- | --- | --- | --- |
|  | IRR (95% CI) | P | IRR (95% CI) | P | IRR (95% CI) | P | IRR (95% CI) | P | IRR (95% CI) | P | IRR (95% CI) | P |
| **GDP per capita** |  |  |  |  |  |  |  |  |  |  |  |  |
| low | 1.33  (1.14, 1.54) | Ref | 1.19  (1.15, 1.23) | Ref | 0.555  (0.487, 0.633) | Ref | 1.07  (0.972, 1.18) | Ref | 1.14  (1.03, 1.27) | Ref | 0.949  (0.870, 1.03) | Ref |
| high | 2.58  (2.17, 3.08) | <0.001 | 2.12  (1.91, 2.37) | <0.001 | 0.650  (0.575, 0.736) | 0.063 | 1.81  (1.53, 2.16) | <0.001 | 1.18  (1.11, 1.26) | 0.645 | 1.03  (0.982, 1.07) | 0.092 |
| **Population** |  |  |  |  |  |  |  |  |  |  |  |  |
| low | 1.14  (0.981, 1.32) | Ref | 1.10  (1.07, 1.14) | Ref | 0.578  (0.513, 0.653) | Ref | 0.907  (0.810, 1.02) | Ref | 1.08  (0.980, 1.18) | Ref | 0.982  (0.922, 1.04) | Ref |
| high | 2.66  (2.27, 3.13) | <0.001 | 1.94  (1.78, 2.13) | <0.001 | 0.630  (0.562, 0.708) | 0.274 | 1.50  (1.33, 1.71) | <0.001 | 1.25  (1.16, 1.34) | 0.013 | 1.06  (1.01, 1.11) | 0.055 |
| **Isolation** |  |  |  |  |  |  |  |  |  |  |  |  |
| low | 2.18  (1.88, 2.53) | Ref | 1.16  (1.10, 1.23) | Ref | 0.623  (0.545, 0.714) | Ref | 1.38  (1.22, 1.57) | Ref | 1.24  (1.16, 1.32) | Ref | 1.11  (1.05, 1.16) | Ref |
| high | 1.44  (1.24, 1.67) | 0.001 | 1.13  (1.07, 1.19) | 0.176 | 0.598  (0.535, 0.668) | 0.633 | 1.09  (0.989, 1.20) | 0.004 | 1.01  (0.916, 1.12) | 0.005 | 0.893  (0.826, 0.960) | <0.001 |
| **Fragmentation** |  |  |  |  |  |  |  |  |  |  |  |  |
| low | 2.04  (1.77, 2.34) | Ref | 1.19  (1.14, 1.24) | Ref | 0.574  (0.506, 0.653) | Ref | 1.31  (1.17, 1.47) | Ref | 1.20  (1.13, 1.27) | Ref | 1.10  (1.03, 1.17) | Ref |
| high | 1.16  (0.998, 1.35) | <0.001 | 1.05  (0.997, 1.11) | <0.001 | 0.573  (0.515, 0.638) | 0.988 | 0.973  (0.877, 1.08) | <0.001 | 1.01  (0.949, 1.08) | 0.001 | 0.977  (0.925, 1.03) | 0.004 |

# Table S6. Incidence rate ratio of dengue for greenness structure indices per interquartile range increase at different levels of urbanization and built environment characteristics.

high, high level of urbanization and built environment characteristics (concentration for 75th value), low, low level of urbanization and built environment characteristics (concentration for 25th value).

Isolation, mean distance to the nearest urban patch within the geographic boundary; Fragmentation, number of urban patches divided by the total area of the geographic unit; IRR, incidence rate ratio; CI, confidence interval.

# Table S7. Sensitivity analyses by changing covariates in the main model.

|  | Main model |  | Extended model |  | Less-adjusted model |
| --- | --- | --- | --- | --- | --- |
|  | IRR (95% CI) |  | IRR (95% CI) |  | IRR (95% CI) |
| Percentage of landscape | 1.78 (1.57, 2.01) |  | 1.78 (1.58, 2.02) |  | 1.78 (1.57, 2.01) |
| Mean patch area | 1.14 (1.10, 1.20) |  | 1.15 (1.10, 1.20) |  | 1.14 (1.10, 1.19) |
| Edge density | 0.606 (0.547, 0.671) |  | 0.606 (0.547, 0.671) |  | 0.608 (0.550, 0.674) |
| Area weighted mean shape index | 1.17 (1.06, 1.29) |  | 1.17 (1.07, 1.29) |  | 1.17 (1.06, 1.29) |
| Aggregation index | 1.19 (1.11, 1.26) |  | 1.19 (1.11, 1.26) |  | 1.19 (1.11, 1.26) |
| Patch cohesion index | 1.03 (0.983, 1.07) |  | 1.03 (0.983, 1.07) |  | 1.03 (0.984, 1.08) |

Main models adjusted for average annual temperature, cumulative annual rainfall, relative humidity, total population, GDP per capita, and urban isolation. Extended models were additionally adjusted for the density of road network. Less-adjusted models did not adjust for relative humidity compared to the main model.

IRR, incidence rate ratio; CI, confidence interval.

There was no significant difference between the results of extended model and less-adjusted model and the results of main model (p values ranges 0.93-0.99).

**3. Supporting Figures**


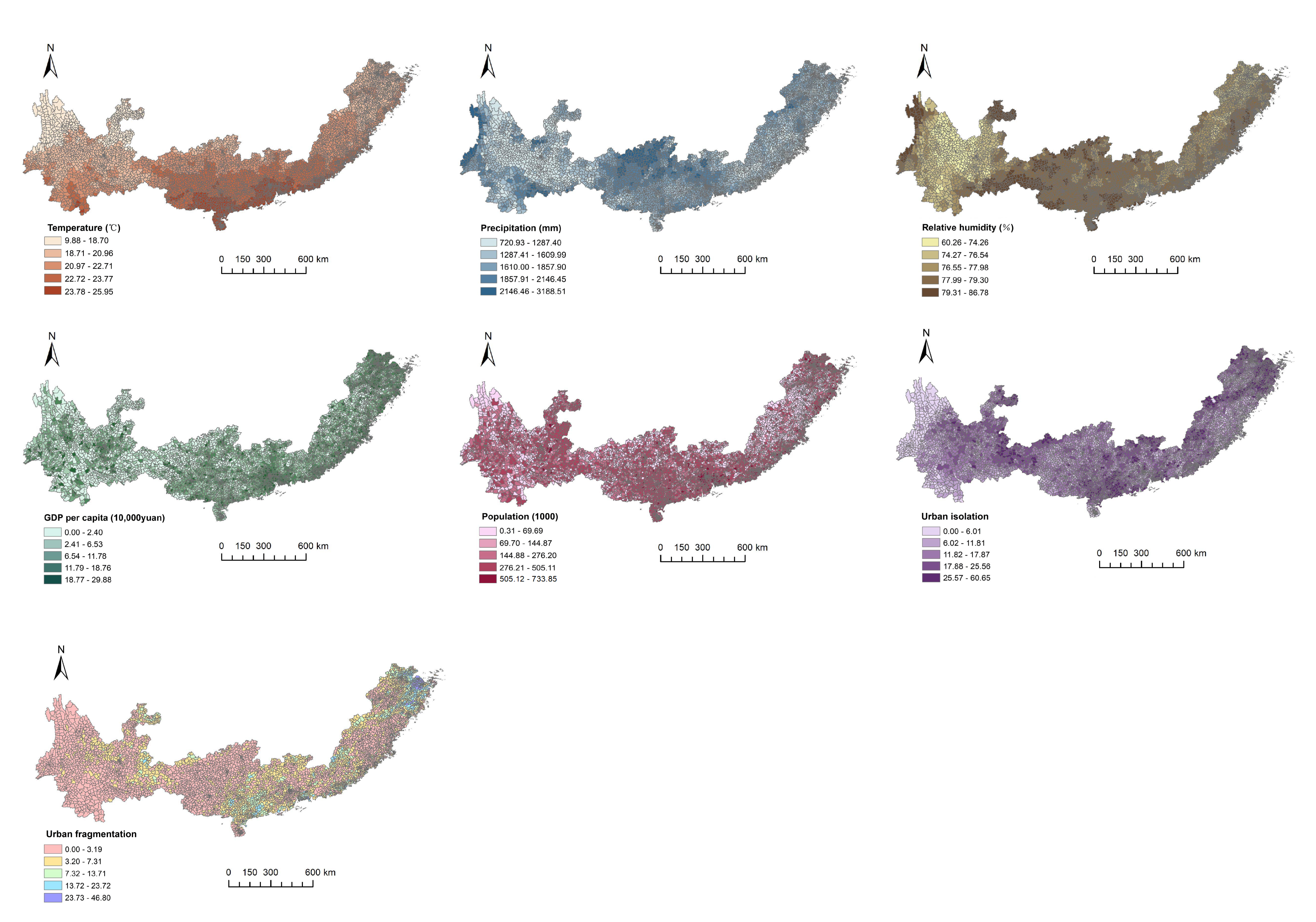


# Figure S1. Spatial distribution of annual mean value of climatic, urbanization, and built environment characteristics of the five provinces in China, 2017-2020.


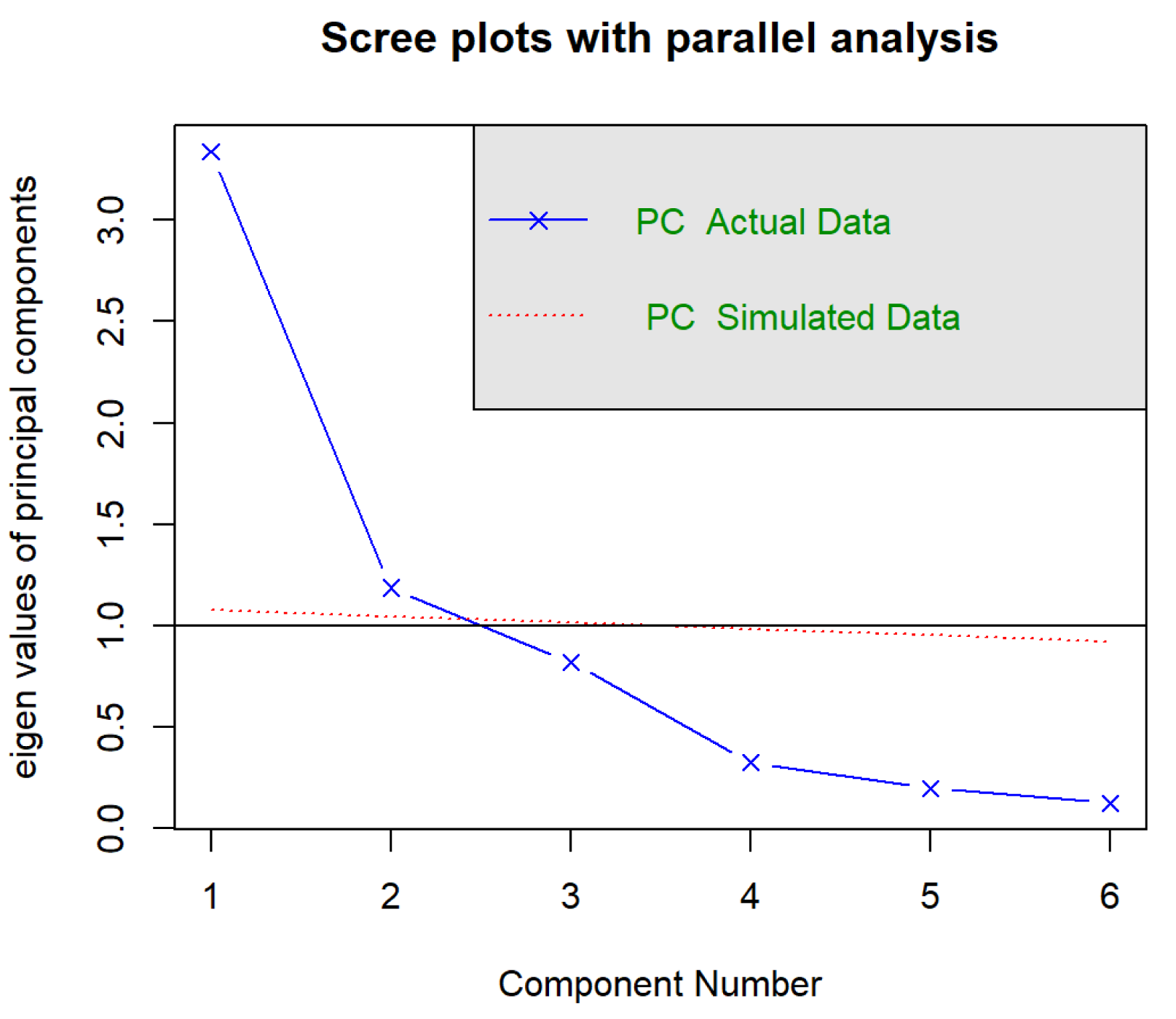


# Figure S2. Gravel diagram of principal components of greenspace component.

# References

1. Ministry of Health of People's Republic of China. Diagnostic criteria for dengue fever (WS 216-2008). 2008. <http://www.nhc.gov.cn/wjw/s9491/200802/38819.shtml>. Accessed 20 Jan 2024.

2. National health commission of the People’s Republic of China. Diagnosis for dengue fever (WS 216-2018). 2018. <http://www.nhc.gov.cn/wjw/s9491/201803/d524df26df28453eada8371dc3565818.shtml>. Accessed 20 Jan 2024.
